# Thermal crosstalk in 3-dimensional RRAM crossbar array

**DOI:** 10.1038/srep13504

**Published:** 2015-08-27

**Authors:** Pengxiao Sun, Nianduan Lu, Ling Li, Yingtao Li, Hong Wang, Hangbing Lv, Qi Liu, Shibing Long, Su Liu, Ming Liu

**Affiliations:** 1 Key Laboratory of Microelectronics Devices and Integrated Technology, Institute of Microelectronics, Chinese Academy of Sciences, Beijing 100029 China; 2 Lab of Nanofabrication and Novel Devices Integration Technology, Institute of Microelectronics, Chinese Academy of Sciences, Beijing 100029 China; 3 School of Physical Science and Technology, Lanzhou University, Lanzhou 730000 China; 4 School of Advanced Materials and Nanotechnology, Key Laboratory of Wide Band Gap Semiconductor Materials and Devices, Xidian University, Xi’an 710071 China

## Abstract

High density 3-dimensional (3D) crossbar resistive random access memory (RRAM) is one of the major focus of the new age technologies. To compete with the ultra-high density NAND and NOR memories, understanding of reliability mechanisms and scaling potential of 3D RRAM crossbar array is needed. Thermal crosstalk is one of the most critical effects that should be considered in 3D crossbar array application. The Joule heat generated inside the RRAM device will determine the switching behavior itself, and for dense memory arrays, the temperature surrounding may lead to a consequent resistance degradation of neighboring devices. In this work, thermal crosstalk effect and scaling potential under thermal effect in 3D RRAM crossbar array are systematically investigated. It is revealed that the reset process is dominated by transient thermal effect in 3D RRAM array. More importantly, thermal crosstalk phenomena could deteriorate device retention performance and even lead to data storage state failure from LRS (low resistance state) to HRS (high resistance state) of the disturbed RRAM cell. In addition, the resistance state degradation will be more serious with continuously scaling down the feature size. Possible methods for alleviating thermal crosstalk effect while further advancing the scaling potential are also provided and verified by numerical simulation.

To satisfy the growing requirements for enormous data densities and nonvolatile storage, new memory technologies are currently attracting much attention due to their significant potential for the replacement of FLASH memory^1^,^2^,^3^,^4^,^5^,^6^,^7^,^8^,^9^,^10^,^11^,^12^. High density 3-dimensional (3D) RRAM crossbar array is one of the major focuses for the new age technology^12^,^13^,^14^,^15^,^16^,^17^. To compete with the ultra-high density 3D NAND FLASH, understanding of reliability mechanisms and scaling potential of 3D RRAM crossbar array during operation is necessary. Thermal crosstalk is one of the most critical effects that should be considered in 3D crossbar array application. The Joule heat generated inside the RRAM device will determine the switching behavior of the device, and for high density memory arrays, the temperature surrounding may lead to a consequent resistance degradation of neighboring devices during cycling. Moreover, due to the crosstalk issue between the adjacent devices, scaling potential of the integrated array under thermal effect must be seriously addressed.

Generally, to suppress the current sneak path, an additional selective component is always required in the crossbar integration^12^,^18^,^19^,^20^,^21^,^22^,^23^,^24^, and 1D1R (one Diode one RRAM) structure is very attractive for 3D cross-point architecture in terms of the vertical stackable ability and the simplicity of the erasing/programing method^24^,^25^,^26^,^27^,^28^. 1D1R storage element usually displays unipolar switching (set and reset operation at the same voltage polarity)^26^,^29^, and the reset process is controlled by Joule heating^26^,^30^. Understanding of programming and reliability mechanisms in unipolar 1D1R crossbar array requires a detailed characterization of the electrical and thermal conduction properties of the memory device. Many researches have been performed in the thermal effects of RRAM^30^,^31^,^32^,^33^,^34^, however, all the previous works were based on an individual device level and neglected the diode device. Thermal effect in 3D RRAM crossbar array is still lacking up to date.

In this work, thermal crosstalk effect in 3D RRAM crossbar array was systematically investigated. It is revealed that the transient thermal effect plays a dominant role in reset process. More importantly, thermal crosstalk phenomena could deteriorate device retention performance and even lead to data storage state failure from LRS (low resistance state) to HRS (high resistance state) of the disturbed RRAM cell. In addition, the resistance state degradation will be more serious with continuously scaling down the feature size. Possible methods for alleviating thermal crosstalk effect are also provided and verified by numerical simulation. The results in this work were computed for unipolar 1D1R crossbar arrays but are likely to be a reference to bipolar RRAM device based arrays as well, due to the thermal nature in the resistive switching process.

## Physical Model Description

**Figure 1:**
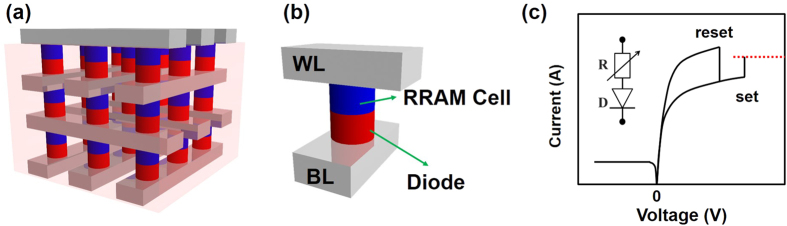
(**a**) Schematic of 3D 1D1R crossbar array structure. (**b**) Schematic of 1D1R storage element which is composed of a RRAM device and a diode connected in series. (**c**) Schematic diagram of typical DC I-V characteristic of the 1D1R element. In this work, voltage is applied to the electrode (WL/BL) that is connected with RRAM cell while keeping the opposite electrode ground for the reset operation.

Figure 1a,b show the schematic diagrams of 1D1R crossbar array structure and 1D1R data storage element which is composed of a RRAM and a diode connected in series, respectively. Figure 1c shows the schematic of typical I-V characteristics including set and reset operations of 1D1R structure^26^. Both set and reset occur at the same voltage polarity. In this full manuscript, voltage is applied to the electrode connected with RRAM while keeping the opposite electrode ground for the set/reset operations. Thermal behavior in a cross-point array can be described through 3D Fourier heat flow equation,


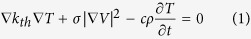


where *k*_*th*_ is the thermal conductivity, *T* is the temperature, *c* is the thermal capacity, *ρ* is the mass density of the materials in the crossbar array system, *V* is the imposed voltage, *t* is time and *σ* is the electric conductivity which empirically reads as


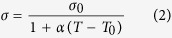


where *α* is the resistivity temperature coefficient and *σ*_0_ is the electric conductivity at room temperature *T*_0_. Word lines (WL) or bit lines (BL) in the top and bottom layers of 3D array system are assumed to connect with ideal heat dissipation packaging structure and keep at room temperature *T*_0_ = 300 K during the calculation, as


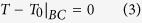


Ni/HfO_2_/Pt RRAM device and Ti/TiO_2_/Pt diode based 1D1R structure and their corresponding electrical parameters in ref. 26 are used as a reference in this work. The insulating material between each 1D1R cell is HfO_2_. The resistive switching behavior of Ni/HfO_2_/Pt RRAM device is widely accepted to via formation/rupture of Ni conductive filament (CF)^35^,^36^. The reset current *I*_*reset*_ is 1.7 × 10^−4^ A. Detailed parameters used in the calculation are listed in Table 1.

## Results and Discussion

**Figure 2:**
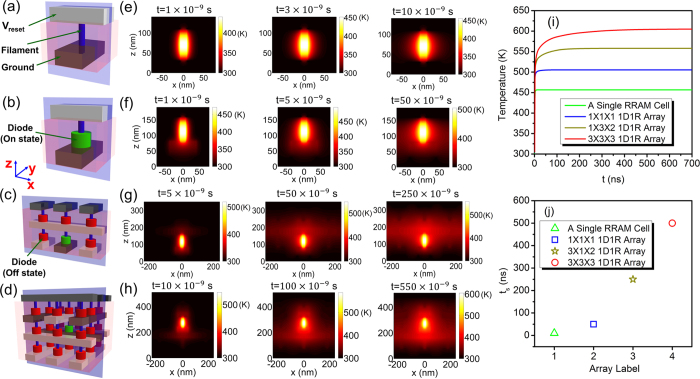
(**a**) Schematic of the structure of an individual RRAM cell. (**b**–**d**) Schematics of the structure of 1D1R crossbar block with 1 × 1 × 1, 3 × 1 × 2 and 3 × 3 × 3 array size, respectively. (**e**,**f**) Calculated temperature evolution maps of the cross-sections (blue planes) in (**a**–**d**). (**i**) Highest temperature evolution in the programmed RRAM device for the 4 selected structure in (**a**–**d**). (**j**) *t*_*s*_ (time that array system consumes to reach thermal steady state) as a function of the array size. The WL/BLs with *V*_*reset*_ are marked in white and the ones being grounded are marked in black. The programmed RRAM cells are connected in series with on-state diodes (marked in green), and the unprogrammed RRAMs are connected with off-state diodes (marked in red).

Firstly, dynamic temperature evolution in crossbar array system was studied. Figure 2 shows the temperature evolution during reset operation for several cross-point arrays with different sizes. Here, all the RRAM cells are in LRS. Figure 2a–d illustrate the schematics of array structures in the simulation, including an individual RRAM cell and 3D RRAM arrays with various sizes from a 1 × 1 × 1 1D1R element to 3 × 3 × 3 block array, respectively. The WL/BLs with *V*_*reset*_ are marked in white and the ones being grounded are marked in black. The programmed RRAM cells are connected with on-state diodes (marked in green), and the unprogrammed RRAMs are connected with off-state diodes (marked in red). At the periphery of the array structures, heat dissipates through the surface of the simulated volume with a typical heat transfer coefficient 10 *W*/*m*^2^*K* of air. Figure 2e–h show the corresponding temperature evolution maps of the cross-section (blue planes in Fig. 2a–d inside the arrays). Figure 2i shows the highest temperature evolution in the programmed RRAM device of the array systems in Fig. 2. Figure 2j shows the corresponding time *t*_*s*_, *t*_*s*_ is the time that array system consumes to reach thermal steady state. From Fig. 2i, one can clearly see that *t*_*s*_ increases with the increase of array size as the heat capacity of the system gets higher. In Fig. 2j, the derived *t*_*s*_ is 50 ns for a 1D1R element, which is much higher than that for the individual RRAM (less than 5 ns) since the diode part could hold certain amount of heat during the heating process. For the 3 × 3 × 3 block array, *t*_*s*_ would be over 500 ns, which is about 10 times as that of a single 1D1R element. 3D 1D1R crossbar array includes masses of devices, hence the thermal effect is highly complex. The peak temperature of steady state also varies remarkably with the array sizes (e.g. 500 K for a single 1D1R device, and 605 K for that in 3 × 3 × 3 array system), since the distances between thermal source (the programmed RRAM cell) and the thermal dissipation boundaries (top/bottom electrodes in this work) are different. For an array with small size, the programmed cell is close to the top/bottom boundary, the generated Joule heat can be easily dissipated to the thermal sink boundaries, hence the corresponding temperature of the cell would be lower. While For an array with large size, the generated Joule heat could hardly dissipate. Finally the accumulated Joule heat would increase the final temperature of array with the large size. Besides that, in a 3D crossbar array with multiple stack layer, the temperature is also very different when the programmed RRAM cell locates in different layers, e.g. in a 3 × 3 × 3 block array, the difference of the final temperature for the programmed RRAM device in different layers could reach about 50 K (as shown in Figure S1-S2, Supporting Information). The typical reset time of RRAM devices usually varies from tens of ns to hundreds of ns^37^,^38^,^39^,^40^,^41^. For a single RRAM device, *t*_*s*_ is much shorter than the typical reset time, i.e. RRAM device could reach steady thermal state far before the reset process is completed, and it is reasonable to use the steady thermal state temperature in device model and simulation. While for dense memory arrays, *t*_*s*_ could be much larger than the typical reset time, which means that the individual RRAM device model based on steady thermal state is not suitable for 3D cross-point array.

**Figure 3:**
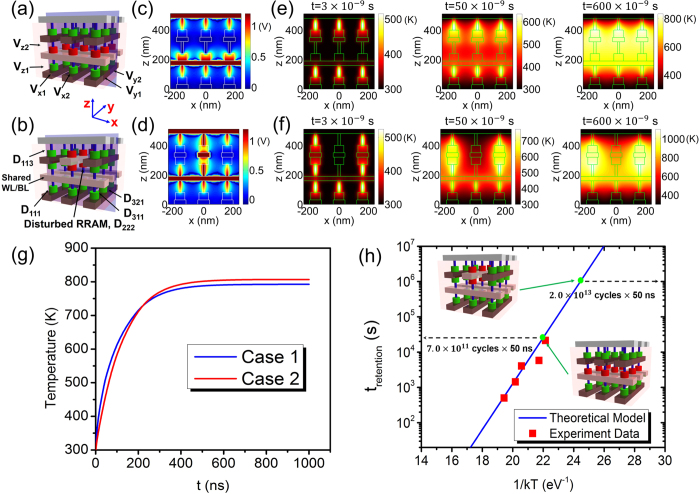
(**a**,**b**) Schematics of the two selected “worst cases” in thermal crosstalk analysis. (**b**) A crossbar array with shared WL/BLs, which could realize parallel erasing/programming at different layers. (**c**,**d**) Potential maps inside the array structures in (**a**,**b**). The WL/BLs imposed with *V*_*reset*_ are marked in white and the ones being grounded are marked in black. The programmed RRAM cells are connected in series with on-state diodes (marked in green), and the unprogrammed RRAMs are connected with off-state diodes (marked in red). The disturbed RRAM cell (labeled as *D*_222_) locates in the center of crossbar array surrounded by multiple programmed RRAM devices. (**e**,**f**) Temperature evolutions of the cross-sections (blue planes in (**a**,**b**)) for the two array structures. (**g**) Highest temperature evolution in the disturbed RRAM cell (*D*_222_) in the two selected “worst cases” array structures. Case 1 and Case 2 correspond to the cases in (**a**,**b**), respectively. (**h**) Arrhenius plot of the measured and modeled retention time (reference from ref. 26 in which the conductive path of the RRAM device is modeled as metallic Ni rich filament) and the evaluation of the device reliability under thermal crosstalk. The disturbed RRAM cell may be failure from LRS to HRS after 7.0 × 10^11^ and 2.0 × 10^13^ consecutive erase/program cycles for the two cases in (**a**,**b**).

From Fig. 2, it is clear that thermal transfer is fast along the WL/BLs and the CFs of RRAMs in both horizontal and vertical directions due to their high thermal transfer ability. In this situation, passive temperature increase in the adjacent RRAM devices would be induced by thermal crosstalk, which may deteriorate device reliability and even lead to failure of disturbed RRAM cells. To evaluate the reliability of the 3D RRAM array under a parasitic thermal crosstalk, we calculated the 3D temperature profiles in a small 3 × 3 × 3 block array consisting of 27 cells with feature size of 80 nm, as shown in Fig. 3a,b.

To understand clearly the thermal crosstalk effect, the temperature profile was calculated for the two “worst cases”. Here all the RRAM cells are in LRS before their resistance change from LRS to HRS by applying a reset pulse in the programmed cells. Figure 3a shows the typical crossbar array structure and Fig. 3b shows the crossbar array with shared WL/BLs^28^, which could realize parallel erasing/programming devices at different stack layers. The programmed RRAM devices are connected in series with on-state diodes (marked in green), and the unprogrammed RRAMs are connected with off-state diodes (marked in red). The disturbed RRAM cell (labeled as *D*_222_) locates in the center of the crossbar array surrounded by multiple programming RRAM devices. In Fig. 3a, thermal crosstalk effect mainly results from the neighboring RRAM devices in vertical direction of different layers. While in Fig. 3b, crosstalk influence results from adjacent RRAM devices in both vertical direction of different layers and horizonal direction within the same layer.

Figure 3c–f show the calculated potential distributions and temperature evolutions of the cross-sections in the 3D array systems, respectively, corresponding to the two selected cases in Fig. 3a,b. Here, periodical boundary condition is adopted at the periphery of the array structures during the simulation as there could be masses of devices within the same stack layer for dense memory arrays. It is found obviously that temperatures in the programmed RRAM devices rise much faster than those in the unprogrammed ones. In addition, temperature in the unprogrammed RRAM region passively increases with increasing time due to the thermal crosstalk effect. Figure 3g illustrates the highest temperature evolution in the disturbed RRAM device (*D*_222_) for the two selected “worst case” array structures. Case 1 and Case 2 correspond to the selected cases in Fig. 3a,b, respectively. The temperature in the disturbed RRAM device increases with time and could ultimately reach 792 K and 807 K, respectively.

Figure 3g shows the Arrhenius plot of the experimental and simulated retention performance of NiO based RRAM device^42^, where the conductive path is treated as metallic Ni filament^30^. Thermal dissolution of the metallic-conductivity filament, which may be induced by the dissipation of conductive elements from the filament, may result in premature loss of the resistance state of RRAM, thus causing a retention reliability concern^43^. Considering the dominant role of transient thermal effect plays during reset operation in 3D crossbar array, transient temperature is adopted to evaluate the thermal crosstalk effect on device reliability. Here, we used a conservative estimation method^44^: assuming the typical reset time *t*_*reset*_ is 100 ns for a standalone 1D1R device. The temperature in the RRAM filament can be converted into retention time *t*_*retention*_ according to the Arrhenius plot in Fig. 3g. The highest temperature *T*_*p*_ at *t* = 50 ns in the disturbed RRAM cell is 523 K and 475 K, as shown in Fig. 3h. Based on the experimental data, the derived *t*_*retention*_ at *T*_*p*_ = 523 K and *T*_*p*_ = 475 K is 3.5 × 10^4^ s and 1.0 × 10^6^ s, respectively. Here, consecutive program/erase operations is conservatively assumed to have similar effect of continuously heating at a constant temperature (*T* = *T*_*p*_), with effective heating time *t*_*h*_ = *t*_*reset*_ − 50 ns for each program/erase cycle. Then the derived *t*_*retention*_ correspond to a sequence of *t*_*retention*_/(*t*_*reset*_ − 50 ns) = 7.0 × 10^11^ and 2.0 × 10^13^ program/erase pulses, respectively (i.e., reasonable cycling expectations for RRAM devices), whereas the disturbed bit is not programmed in the same time frame. In other words, the thermal crosstalk deteriorates the LRS retention reliability and the disturbed RRAM cell may be failure from LRS to HRS after 7.0 × 10^11^ and 2.0 × 10^13^ consecutive program/erase cycles for the 2 cases in Fig. 3. Here, self-heating effect due to the application of a set pulse to the programmed bit is neglected since the temperature is much lower than that in the reset operation due to the lower programming current. It should be mentioned that this is a relative conservative estimation method due to the transient nature of the Joule heating effect in 3D RRAM array (in order to give an intuitive and feasible estimation of thermal crosstalk, in this work, the influence of interval time between each program/erase pulse was neglected for the sake of simplicity), and the actual case in the very large scale 3D array could be more complex.

**Figure 4:**
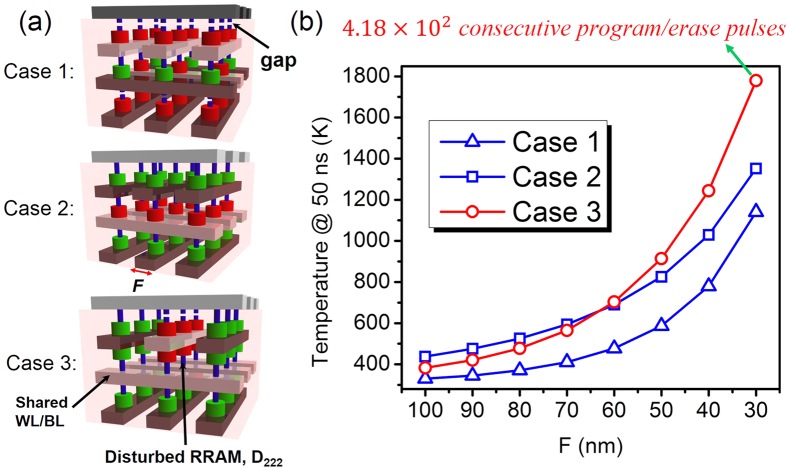
Schematic diagrams of the “worst cases” selected in scaling analysis. (**b**) Highest temperature at *t* = 50 ns in the disturbed RRAM device with feature size *F* scaling down from 100 nm to 30 nm node for the 3 selected cases in (**a**). RRAM in HRS (with 5 nm gap in the CF) are also included. Case 1 and Case 2 adopt the typical crossbar structure (blue lines in (**b**)). Case 3 adopts the structure with shared WL/BL, which could realize parallel erasing/programming at different layer (red line in (**b**)). The WL/BLs imposed with *V*_*reset*_ are marked in white and the ones being grounded are marked in black. The programmed RRAM cells are connected in series with on-state diodes (marked in green), and the unprogrammed RRAMs are connected with off-state diodes (marked in red). The disturbed RRAM cell (labeled as *D*_222_) locates in the center of crossbar array surrounded by multiple programmed RRAM devices.

Understanding the thermal crosstalk between neighboring RRAM devices is a critical step to understand the scaling potential and performance tradeoffs associated with miniaturization. In scaling analysis, we use the same method in Fig. 3h to evaluate thermal crosstalk on LRS reliability. Figure 4 shows the highest temperature at *t* = 50 ns in the disturbed cell (*D*_222_) for 3 selected “worst cases” (Fig. 4a) with feature size *F* scaling down from 100 nm to 30 nm node. RRAM devices in HRS (with 5 nm gap in the CF) are also included. Here, the crossbar structure in Case 1 and Case 2 is similar (blue lines in Fig. 4b). In Case 1, the crosstalk results from the neighboring RRAM devices in horizontal direction. While in Case 2, the crosstalk results from the neighboring RRAM cells in different layers in vertical direction. The array structure in Case 3 adopts shared WL/BLs (shown in Fig. 4a), which could realize parallel programming/erasing at different layer^28^ (red line in Fig. 4b). One can see in Fig. 4b that the temperature in the disturbed RRAM device increases with *F* scaling down for the 3 selected cases. For the array structure in Case 1 and Case 2, thermal crosstalk from the neighboring CFs within the same layer (Case 1) is smaller than that in vertical direction (Case 2) in the range of 100 nm to 30 nm node, and thermal crosstalk of Case 3 will be stronger than that in Case 2 at 62 nm node. The temperature in the disturbed RRAM filament could even reach 1780 K for Case 3 at 30 nm node, which corresponds to 4.18 × 10^2^ consecutive program/erase cycles (the disturbed cell will be failure from LRS to HRS after 4.18 × 10^2^ consecutive program/erase cycles due to thermal crosstalk).

It should be noted that the values in this work have been performed for the purpose of scaling analysis, and although reasonable, they should not be considered as mandatory values for the 1D1R crossbar array scaling roadmap.

**Figure 5:**
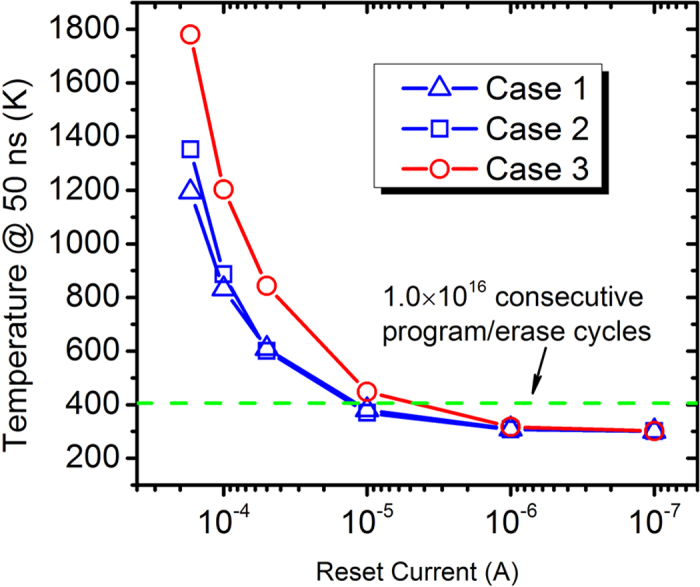
Highest temperature at *t* = 50 ns in the disturbed RRAM device as a function of reset current *I*_*reset*_ for *F* = 30 nm. Case 1-Case 3 correspond to the 3 “worst cases” in Fig. 4, respectively. With the decrease of *I*_*reset*_ from 1.7 × 10^−4^ A to 1.0 × 10^−7^ A, temperature in the disturbed RRAM filament decreases remarkably. Using the same evaluation method in Fig. 3h, the storage state of the disturbed RRAM could stand 1.0 × 10^16^ consecutive program/erase cycles (endurance requirement of DRAM devices) with temperature at *t* = 50 ns equals to 406 K, which corresponds to *I*_*reset*_ = 1.2 × 10^−5^ A, *I*_*reset*_ = 1.2 × 10^−5^ A and *I*_*reset*_ = 4.7 × 10^−6^ A (reasonable expectations of unipolar RRAM devices^45^,^46^) for the 3 “worst cases”, respectively.

Decreasing the reset current *I*_*reset*_ could effectively alleviate thermal crosstalk. Figure 5 shows the calculated highest temperature at *t* = 50 ns in the disturbed RRAM device as a function of *I*_*reset*_ for *F* = 30 nm. In the figure, the values were calculated for 3 “worst cases” in Fig. 4. With the decrease of *I*_*reset*_ from 1.7 × 10^−4^ A to 1.0 × 10^−7^ A, temperature in the disturbed RRAM filament decreases remarkably. Using the same evaluation method in Fig. 3h, the storage state of the disturbed RRAM could stand 1.0 × 10^16^ consecutive program/erase cycles (endurance requirement of DRAM devices) with temperature at *t* = 50 ns equals to 406 K, which corresponds to *I*_*reset*_ = 1.2 × 10^−5^ A, *I*_*reset*_ = 1.2 × 10^−5^ A and *I*_*reset*_ = 4.7 × 10^−6^ A (reasonable expectations of unipolar RRAM devices^45^,^46^) for the 3 “worst cases”, respectively.

Besides that, to continue miniaturization, a simple cycle-rehabilitate technique can also be used: erasing and reprogramming the LRS of RRAM cells in the array system after a certain operation cycles *c*_*r*_ (making sure that after *c*_*r*_ cycles, the deteriorated LRS can still be used to distinguish HRS and LRS). Using this method, the resistance of RRAM devices in LRS deteriorated by thermal crosstalk could be rehabilitated by the reprogram operation, and the scaling potential of crossbar array can be further advanced.

In summary, the dominant role of transient thermal effect on the reset mechanisms was demonstrated, thermal crosstalk on the RRAM retention property and the scaling potential of 3D RRAM array under thermal effect were analyzed in detail based on the numerical simulation in this work. According to theoretical analysis, it is revealed that 1) the individual RRAM device models based on steady state thermal effect may not be applicable in 3D device crossbar array; 2) thermal crosstalk phenomena could deteriorate device retention performance and even lead to disturbed RRAM component failure from LRS to HRS, especially with continuously scaling down the feature size; 3) decreasing the reset current and adopting the cycle-rehabilitate technique could alleviate thermal crosstalk phenomena for LRS retention characteristics of the array while further advancing the scaling potential.

## Additional Information

**How to cite this article**: Sun, P. *et al.* Thermal crosstalk in 3-dimensional RRAM crossbar array. *Sci. Rep.* **5**, 13504; doi: 10.1038/srep13504 (2015).

## Figures and Tables

**Table 1:** Parameters used in the simulation.

Parameter	Value	Parameter	Value	Parameter	Value
*r*_*cf*_^26^,^30^	8 *nm*	*r*_*diode*_	40 *nm*	*h*_*line*_^26^	30 *nm*
*h*_*cf*_^26^	80 *nm*	*h*_*diode*_^26^	50 *nm*	*w*_*line*_	80 *nm*
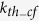 47	22 *W*/(*mK*)	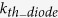 48	11.7 *W*/(*mK*)	*c*_*line*_^47^	445 *J*/(*kgK*)
*c*_*cf*_^47^	445 *J*/(*kgK*)	*c*_*diode*_^49^	710 *J*/(*kgK*)	*ρ*_*line*_^47^	8.9 × 10^3^ *kg*/*m*^3^
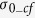 26,30	1.23 × 10^5^ *S*/*m*	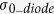 26	3.07 × 10^3^ *S*/*m* & 5.0 × 10^−2^ *S*/*m*	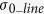 26	1.23 × 10^5^ *S*/*m*
*α*_*cf*_^36^	0.0014	*ρ*_*diode*_^47^	4.17 × 10^3^ *kg*/*m*^3^	*V*^26^	1.2 *V*
*ρ*_*cf*_^47^	8.9 × 10^3^ *kg*/*m*^3^	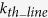 47	22 *W*/(*mK*)	*cim*^47^	286 *J*/(*kgK*)
*ρ*_*im*_^47^	9.68 × 10^3^ *kg*/*m*^3^	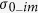 26	7 × 10^−7^ *S*/*m*	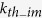 50	0.5 *W*/(*mK*)

*r* is radius, *h* is thickness, *k*_*th*_ is thermal conductivity, *c* is heat capacity, *σ*_0_ is the reference electric conductivity and *w* is width. The subscripts *cf*, *diode*, *line* and *im* denote CF (conductive filament), diode, WL/BL component and insulating material between 1D1R cells, respectively. *V* is reset voltage, and 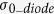 list two values which correspond to the on-state value and off-state value of the diode device.
